# Smoking is Associated with Reduced Leptin and Neuropeptide Y Levels and Higher Pain Experience in Patients with Fibromyalgia

**DOI:** 10.1155/2014/627041

**Published:** 2014-08-14

**Authors:** Maria I. Bokarewa, Malin C. Erlandsson, Jan Bjersing, Mats Dehlin, Kaisa Mannerkorpi

**Affiliations:** Department of Rheumatology and Inflammation Research, Institute of Medicine, Sahlgrenska Academy at the University of Göteborg, P.O. Box 480, 405 30 Göteborg, Sweden

## Abstract

Smoking deregulates neuroendocrine responses to pain supporting production of neuropeptide Y (NpY) by direct stimulation of nicotinic receptors or by inhibiting adipokine leptin. Present study addressed the effect of cigarette smoking on adipokines and pain parameters, in 62 women with fibromyalgia (FM) pain syndrome with unknown etiology. Pain was characterized by a visual analogue scale, tender point (TP) counts, pressure pain threshold, and neuroendocrine markers NpY and substance P (sP). Levels of IGF-1, leptin, resistin, visfatin, and adiponectin were measured in blood and cerebrospinal fluid. Current smokers (*n* = 18) had lower levels of leptin compared to ex-smokers (*n* = 25, *P* = 0.002), while the expected NpY increase was absent in FM patients. In smokers, this was transcribed in higher VAS-pain (*P* = 0.04) and TP count (*P* = 0.03), lower pain threshold (*P* = 0.01), since NpY levels were directly related to the pain threshold (rho = 0.414) and inversely related to TP counts (rho = −0.375). This study shows that patients with FM have no increase of NpY levels in response to smoking despite the low levels of leptin. Deregulation of the balance between leptin and neuropeptide Y may be one of the essential mechanisms of chronic pain in FM.

## 1. Introduction

Adipose tissue is recognized as an active endocrine organ homing several metabolic and immunologically active cell types including fat cells, macrophages, endothelial cells, and stromal cells. Adipose tissue releases a vast number of biologically active growth factors, cytokines, chemokines, and hormone-like molecules also called adipokines [1, 2]. Adipokines act as both extracellular mediators and intracellular sensors that are important regulators of carbohydrate metabolism, insulin sensitivity, and lipid metabolism controlling body weight. Adipokines have a profound influence on the regulation of inflammation and immune responses [3]. The most studied adipokines to date, leptin, visfatin, and resistin, are considered to be proinflammatory, while adiponectin has been described to have anti-inflammatory properties. Adipokines interact with their specific receptors (leptin receptors a and beta, adiponectin receptors AdipoR1 and AdipoR2) or utilize the insulin/IGF-1 receptor (IGF-1R) activating JAK/STAT, MAP-kinase, and NF-kB signaling pathways and triggering production of endothelial growth factors, proinflammatory cytokines such as TNFa, IL-6, IL-1b.

It has become obvious that adipokines are transported across the blood-brain barrier to the central nervous system and have prominent role in the central control of body functions. Leptin and adiponectin enter brain through the circumventricular organs interacting with the receptors in the hypothalamic nuclei, hippocampus, cortex, cerebellum, and spinal cord regulating neuronal function [4]. Leptin and resistin have been implied in pain processing in patients with spinal cord injury, myocardial infarction and chronic angina pectoris, and knee osteoarthritis [5–7]. Leptin-deficient mice have low pain sensitivity [8]. The molecular mechanism of pain studied in animal models revealed the ability of leptin and resistin to activate hypothalamic neurons changing phosphorylation of central enzymatic targets acetyl-CoA carboxylase, 5′AMP-activated protein kinase, STAT3 and production of neuropeptide Y, and agouti-related peptide [9–12].

Tobacco smoking remains the leading prevalent cause of morbidity and mortality worldwide.

Epidemiological studies show that cigarette smoking is associated with increased chronic pain [13]. Cigarette smokers are overrepresented among persons with pain relative to the general population [14–16]. Pain affects the functional capacity of smokers predicting early retirement [17, 18]. The existing mood disorders as depression and anxiety, personality disorders, and substance use disorders together with social environmental factors social support, occupational functioning, access to health care, and education play an important role in the cause, course, and outcomes of both pain and smoking [19]. Smoking has been viewed as a risk factor for chronic pain [13] and a pain coping strategy in persons with chronic pain [17]. The mechanisms that underlie the apparent negative long-term effects of smoking and exposure to nicotine remain unclear. Nicotine inhibits the activation of opioid and serotoninergic systems, and prolonged exposure to low levels of nicotine results in upregulation of nicotinic acetylcholine receptors in both humans and experimental models [20]. Chronic exposure to nicotine results in nicotine tolerance and increased hyperalgesia. In the experimental setting, the induced hyperalgesia is associated with accumulation of proinflammatory substances and activation of microglia [21, 22].

Fibromyalgia (FM) is a chronic widespread pain syndrome with an estimated prevalence of 2–4% [23, 24]. The core symptoms in patients with fibromyalgia include multifocal pain, tenderness, fatigue, and sleep disturbances [25, 26]. Pain has serious consequences in these patients, as it affects work capacity, relationships, and mood [27]. Patients with fibromyalgia consistently report low quality of life [28], and annual socioeconomic costs of FM are comparable with rheumatoid arthritis [29]. The pathogenesis of pain underlying FM remains unclear, since the condition is not associated with any specific analytical, radiological, or histological findings [30]. Augmented pain and sensory processing are considered the major feature of FM [31]. Recent neuroimaging studies of FM patients showed an increased activity in brain regions that encode the sensory intensity of external stimuli [32]. Several pathogenic processes have been proposed to explain the development of central hypersensitization in FM and include dysfunction of the autonomic nerve system, the neuroendocrine metabolism with abnormalities in the hypothalamic-pituitary-adrenal axis, and immunological activation resulting in a proinflammatory state. In FM, smoking has been outlined among negative factors related to modulating clinical signs and disease progression together with trauma, emotional stress, and infection [26]. Similarly, smokers in FM patients had higher pain sensitivity [33, 34] and higher number of tender points [33] in comparison to nonsmokers.

In the present study we assess the role of adipokines in smoking related changes of clinical and neuroendocrine parameters of pain in patients with FM. We show that patients with FM have no increase of NpY levels in response to smoking despite the low levels of leptin. Deregulation of balance between leptin and neuropeptide Y may be one of the essential mechanisms of chronic pain in FM.

## 2. Materials and Methods

### 2.1. Subjects

The study consisted of 62 patients with FM as defined by the ACR 1990 criteria (a history of long-lasting generalized pain and pain in at least 11 of 18 tender points when examined by manual palpation) [24]. The criteria for inclusion were women with FM that were aged 20–60 years, a willingness to participate in the clinical examinations that included algometry, and sampling of blood by venipuncture, and a willingness to be interviewed about their pain and fatigue. The subjects were also asked to submit cerebrospinal fluid for examination; however this was not a criterion for inclusion. The criteria for exclusion were inability to speak or read Swedish language, presence of other severe somatic or psychiatric diseases, or unwillingness to participate in the study.

### 2.2. Clinical Measurements

Pressure pain thresholds were measured in kPa, using an algometer (Somedic Production AB, Sollentuna, Sweden) with pressure rate increases of 50–60 kPa/s, as previously described [35]. Pain thresholds were measured in two tender points (the upper and lower extremities). The mean values of the pain thresholds were calculated, with a higher value indicating better health. Pain/pain intensity were rated on a visual analogue scale (VAS, 0–100 mm) using the Fibromyalgia Impact Questionnaire (FIQ) [36]. These VAS measurements reflect subjective estimations of overall pain intensities during the last week. A higher score indicates more severe pain. Evaluations of 18 standard tender points (TP) were also performed [24]. The TP counts, ranging from 0 to 18, were calculated and used as estimations of pain distribution. Body mass index (BMI), calculated from a person's weight and height, was used to determine the amount of body fat.

### 2.3. Blood and Cerebrospinal Fluid (CSF) Sampling

Serum samples were acquired from all 62 patients by venipuncture of the cubital vein. Of these, 32 patients provided cerebrospinal fluid (CSF) samples, which were collected by lumbar punctures of the L3/L4 interspace. The collected blood and CSF samples were centrifuged at 800 ×g for 10 min, aliquoted, and stored frozen at –70°C until use.

### 2.4. Laboratory Analyses

Biological markers were analyzed by sandwich enzyme-linked immunosorbent assays (ELISAs) using a pair of specific antibodies for human adiponectin (DY1065, 62 pg/mL), human leptin (DY398, 31 pg/mL), human resistin (DY1359, 10 pg/mL), and human free bioactive IGF-1 (DY291, 4 pg/mL) which were all purchased from RnD Systems (Minneapolis, MN, USA). Assays specific for human visfatin (AG-45A-0006TP-KI01; 125 pg/mL) were purchased from Adipogen Inc. (Incheon, South Korea). Neuropeptide Y (NpY) and substance P were measured by fluorescent EIA kits (Phoenix Pharmaceuticals Inc., Burlingame, CA, USA). All assays were performed according to the instructions of the manufacturers. Ordinary colorimetric ELISAs were read with a Spectramax 340 from Molecular Devices (Sunnyvale, CA, USA) and fluorescent ELISAs were read with a Mithras LB940 from Berthold Technologies (Bad Wildbad, Germany).

### 2.5. Ethics

The Ethical Committee of the Sahlgrenska University Hospital approved the study (220-09). Written and verbal information was given to all patients and written consent was obtained from all patients. The trial is registered at ClinicalTrials.gov (NCT00643006).

### 2.6. Statistical Analysis

Descriptive data are presented as the median, the interquartile range, the number, and the percentage. Correlation between variables was examined by the Pearson's correlation coefficient. The pain intensity was dichotomized using the tender point counts, smoking, and BMI. Difference between groups was assessed using the Mann-Whitney* U* test. Sensitivity and specificity of calculations were performed using 2 × 2 table analysis. Univariate analysis of the association between pain and other serological variables was performed. All tests were two-tailed and conducted with 95% confidence. Statistical analyses were performed using StatView and SPSS v. 19.

## 3. Results and Discussion

### 3.1. Adipokines and Pain

Serum and CSF levels of four major adipokines (visfatin, leptin, resistin, and adiponectin) were measured in the studied cohort of FM patients. Leptin levels in the CSF were significantly higher than levels in serum, while the levels of adiponectin were lower (Table 2).

Leptin is known as the main regulator of hunger and body weight by passing across the blood-brain barrier and modulating activity of neurons in the arcuate nucleus of hypothalamus stimulating production of appetite-suppressing neuropeptides POMC and CART and inhibiting transcription and release of appetite-inducing peptides NpY and agouti-related protein [37]. Indeed, serum and CSF levels of leptin were correlated to BMI across all groups (rho = 0.576, *P* < 0.0001, and rho = 0.374, *P* = 0.0072, resp.). Only smokers had correlation between BMI and the serum levels of resistin (rho = 0.571, *P* = 0.0013) and visfatin (rho = −0.535, *P* = 0.022).

Leptin has been recently identified as an essential indicator of pain sensitivity in animal models and in clinical observational studies. It is locally produced in the perineural adipose tissue in response to neuropathic pain and the mice deficient in leptin have low pain sensitivity [8]. In the present study, pain was measured by VAS, TP counts, and pain threshold (algometry) (Table 1). The univariate analysis showed that the levels of leptin were inversely related to VAS-pain in serum (rho = −0.293, *P* = 0.039) and in CSF (rho = −0.332, *P* = 0.068). Serum levels of resistin were inversely correlated to VAS-pain (rho = −0.35, *P* = 0.001) and this correlation was stronger in nonsmokers (rho = −0.452, *P* = 0.052). CSF levels of resistin were inversely correlated to TP counts (rho = −0.33, *P* = 0.05). Consistent with our findings, the levels of leptin have been previously reported to correlate with pain in patients with spinal injury [38], acute coronary syndrome [39], and knee osteoarthritis [40, 41]. Resistin and visfatin have physiological functions that are the opposite of leptin, as they reduce insulin sensitivity [42, 43] and attenuate hypothalamic leptin signaling [44]. In the studied FM patients, we observed no difference in resistin levels between the smoking groups, and no connection to NpY levels was found.

The modulation of pain by leptin is suggested to occur in the thalamus through the NpY-dependent mechanism [45, 46]. Injection of leptin inhibits postsynaptic release of NpY and enhances pain sensitivity [11]. The antinociceptive and antihyperalgesic effects are one of multiple properties of NpY, the most abundant neuropeptides in the central and peripheral nervous systems which is involved in the generation of new neurons, survival, and functional remodeling of brain cells, feed circuits, and plastic adaptation to behavioral responses (reviewed in [47]).

### 3.2. Adipokines and Medications

Most of the studied patients received analgesics (14 patients, 22.5%), antidepressants (6 patients, 9.7%), or a combination of those two groups of drugs (37 patients, 59.7%), while the remaining 5 patients (8%) had no medication for FM. The comparison of pain evaluation revealed no significant differences in VAS, TP counts, and pain threshold between the FM patients of the four groups indicated above. To study the effect of medications on the levels of adipokines, we compared the groups having a combination of analgesics and antidepressant drugs with the FM patients having no medication. The serum levels of leptin and NpY were 27% higher in the treated patients (leptin, ng/mL, 32.8 [17.2–46.1], NpY, ng/mL, 135 [87–199]) compared to those having no medication (leptin, ng/mL, 20.6 [11.5–39.6], NpY, ng/mL 105 [73.4–138]); however, these differences reached no statistical significance. The levels of resistin, adiponectin, visfatin, and IGF-1 showed no differences between the treated and nontreated FM patients.

### 3.3. Smoking in Leptin-NpY Balance

**Figure 1:**
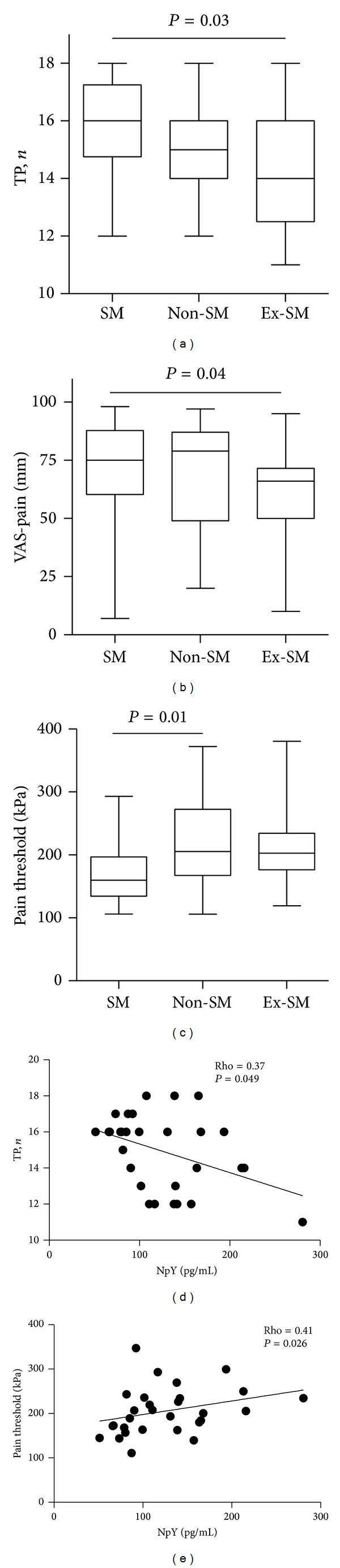
Clinical measurements of pain in patients with fibromyalgia. By smoking habits, the patients were assigned as smokers (SM, *n* = 18), ex-smokers (Ex-SM, *n* = 25), and nonsmokers (non-SM, *n* = 19). (a) Pain distribution was evaluated in 18 standard tender points (TP). (b) Pain intensity was measured by a visual analogue scale (VAS, 0–100 mm). (c) Pressure pain threshold was measured using an algometer. Plots indicate median, interquartile range, and *P* values. The comparison between the groups was done by Kruskal-Wallis with Dunn's post hoc test. Scatterplots show correlation of serum levels of neuropeptide Y (NpY) with TP number (d) and with pressure pain threshold values (e). The Spearman's correlation test was applied.

Stress-related analgesia and reduced pain perception are described in smokers being more pronounced in women [48]. These properties of smoking are used by patients with FM as a pain coping strategy [17]. In the present study, we observed a gradually increasing prevalence of smokers in parallel with increasing TP counts (Figure 1).

Smoking is reported to deregulate neuroendocrine responses to pain by supporting production of NpY [48]. In animal models smoking supports NpY production by direct activation of the a7 and a4b2 nicotinic receptors in the hypothalamus [49] or by regulation of leptin levels in adipose tissue [50]. In FM patients, smokers had lower levels of leptin and visfatin compared to ex-smokers and nonsmokers (Table 2). We could observe no effect of smoking on the levels of adiponectin and resistin. These findings are in agreement with previous observations that smoking men have low serum levels of leptin and a significant suppression of the leptin gene transcription in adipocytes [51–53]. Interestingly, a negative effect of smoking on leptin is transitory in nature. It decreased shortly after smoking [51] and was restored after the cessation of smoking [54, 55]. Smoking had no effect on the levels of adiponectin and resistin. These findings are in agreement with previous reports that smokers have low serum levels of leptin, which is restored shortly after the cessation of smoking [36, 54, 55].

The serum and cerebrospinal fluid (CSF) levels of NpY and substance P were similar in the current smokers, ex-smokers, and nonsmokers (Table 2). Thus, the levels of NpY remained low in the FM smokers and indicated that the reciprocal connection between smoking and NpY was lost and the low levels of leptin provided no increase in NpY levels in patients with FM. These blunt NpY levels in FM smokers could explain high pain parameters.

The current smokers (*n* = 18) had a lower pressure pain threshold compared to nonsmokers (*n* = 19; kPa: 160 [134–197] versus 206 [167–273], *P* = 0.01) and to ex-smokers (*n* = 25; kPa: 203 [176–234], *P* = 0.01), and higher VAS-pain (mm: 75 [60–83] versus 66 [50–72], *P* = 0.04) and TP counts (16 [14.7–17.2] versus 14 [12.5–16.0], *P* = 0.03) when compared to ex-smokers. The clinical measurements of pain correlated to the serum levels of NpY (TP, rho = −0.375, *P* = 0.049; pain threshold, rho = 0.414, *P* = 0.026), while no such correlation was found in the serum levels of substance P. This allowed suggesting that the pain alleviating effects of NpY are observed in the studied FM patients, since the levels of NpY had an inverse correlation to the TP counts and positive correlation to pressure pain threshold, indicating that sensitivity to NpY was not reduced in FM patients. Our findings are consistent with the previously shown inability of FM patients to response pain by adequate production of NpY and altered NpY release [56, 57].

### 3.4. Smoking and IGF-1 

Smoking inhibits IGF-1 levels [58–61]. We observed that FM smokers had low levels of bioactive IGF-1. Furthermore, the levels of IGF-1 were decreased in current and ex-smokers when compared to FM patients that had never smoked. This suggests that smoking has longstanding inhibitory effect on IGF-1 levels. Dysfunction of the GH/IGF-1 axis has been proposed as one of the biological mechanisms that induce widespread pain in FM patients [62, 63]. IGF-1 levels were directly related to NpY levels in CSF (rho = 0.39, *P* = 0.030) and inversely related to fatigue (rho = −0.263, *P* = 0.038), and to serum levels of visfatin (rho = 0.336, *P* = 0.007).

The molecular mechanisms of IGF-1 suppression by nicotine are now quite understood. The levels of IGF-1 are connected with the function of the hypothalamus and IGF-1 may be locally produced in the accurate nucleus. The injection of leptin [64] and the inhibition of NpY [65] are associated with increase of IGF-1 production. In the studied FM patients, the levels of IGF-1 and leptin showed correlation to the CSF levels of NpY indicating a potential connection between disturbances in NpY regulation and low levels of IGF-1 in FM.

## 4. Conclusions

To summarize, patients with FM have low levels of neuropeptide Y. Smoking is associated with low levels of leptin, which is expected to increase NpY levels and to alleviate pain. However, this does not occur in FM and smoking is associated with higher VAS-pain and lower pain threshold. Deregulation of balance between leptin and neuropeptide Y may be one of essential mechanisms of chronic pain in FM.

## Figures and Tables

**Table 1:** Clinical and demographic characteristics of patients with fibromyalgia (*n* = 62).

Age, years	51 [46.2–55.8]
Duration of symptoms, years	10 [8–15]
Pain (VAS, mm)	71 [57–83]
Fatigue (VAS, mm)	82 [67–92]
TP, *n*	15 [13.2–16]
12-13, *n* (%)	16 (26)
14–16, *n* (%)	33 (53)
17-18, *n* (%)	13 (21)
Pain threshold, kPa	199 [159–236]
Smoking habits:	
smokers, *n* (%)	18 (29)
ex-smokers, *n* (%)	25 (40)
Smoking cessation, years	13 [6–17]
Nonsmokers, *n* (%)	19 (31)
BMI, kg/m^2^	28.1 [26.1–30.5]
Medication:	
analgesics, NSAID, *n* (%)	51 (82)
antidepressive, sedative, sleep, *n* (%)	41 (66)

TP, 18 standard tender points. Median and interquartile range are indicated.

**Table 2:** Levels of adipokines and IGF-1 in the blood and cerebrospinal fluid of smokers and nonsmokers.

		Current smokers Blood, *n* = 18 CSF, *n* = 8	Nonsmokers Blood, *n* = 19 CSF, *n* = 11	Ex-smokers Blood, *n* = 25 CSF, *n* = 12
Leptin, ng/mL md [IQR]	Blood	19.4 [11.7–28.7]^££^	28.8 [16.4–48.0]	36.8 [28.0–55.7]
	CSF	104 [91–151]	165 [114–225]	178 [104–244]
Resistin, ng/mL md [IQR]	Blood	14.0 [12.2–17.9]	14.6 [13.2–17.2]	16.5 [14.0–23.8]
	CSF	18.7 [11.7–32.5]	0.0 [0.0-0.0]	24.3 [0.0–32.7]
Adiponectin, *µ*g/mL md [IQR]	Blood	4.6 [3.7–14.2]	4.8 [3.0–7.3]	4.9 [2.7–9.3]
	CSF	0.97 [0.75–1.25]	0.77 [0.64–0.96]	0.86 [0.71–1.03]
Visfatin, ng/mL md [IQR]	Blood	1.32 [1.11–1.65]∗	1.91 [1.34–2.94]	1.33 [1.17–1.72]
	CSF	below det. limit	below det. limit	below det. limit
IGF-1, ng/mL md [IQR]	Blood	2.7 [2.0–4.3]∗	4.8 [4.1–8.8]^$$^	2.5 [1.6–4.2]
	CSF	below det. limit	below det. limit	below det. limit
NPY, pg/mL md [IQR]	Blood	117 [95.9–139]	86.3 [78.5–115]	142 [85.6–180]
	CSF	36.5 [23.3–49.6]	24.5 [16.0–36.9]	29.5 [16.8–36.3]
Substance P, pg/mL md [IQR]	Blood	8.4 [4.3–17.4]	22.0 [9.4–30.8]	16.0 [4.3–46.0]
	CSF	6.3 [2.3–8.8]	5.1 [2.2–6.9]	3.2 [1.0–5.8]

IGF-1, insulin-like growth factor 1. Median, interquartile range, and *P* values (Kruskal-Wallis with Dunn's post hoc) are indicated.

∗Current smokers versus non-smokers *P* < 0.05, ^$$^non-smokers versus ex smokers, *P* < 0.01, ^££^current smokers versus ex smokers *P* < 0.01.

## Abbreviations

FM: Fibromyalgia
GH: Growth hormone
IGF-1: Insulin-like growth factor 1
BMI: Body mass index
VAS: Visual analogue scale
TP: Tender point
NSAID: Nonsteroidal anti-inflammatory drug
IQR: Interquartile range.
